# Achieving Theory–Experiment Parity for Activity and Selectivity in Heterogeneous Catalysis Using Microkinetic Modeling

**DOI:** 10.1021/acs.accounts.2c00058

**Published:** 2022-04-20

**Authors:** Wenbo Xie, Jiayan Xu, Jianfu Chen, Haifeng Wang, P. Hu

**Affiliations:** † School of Chemistry and Chemical Engineering, The Queen’s University of Belfast, Belfast BT9 5AG, U.K.; ‡ Key Laboratory for Advanced Materials, Centre for Computational Chemistry and Research Institute of Industrial Catalysis, East China University of Science and Technology, Shanghai 200237, P. R. China

## Abstract

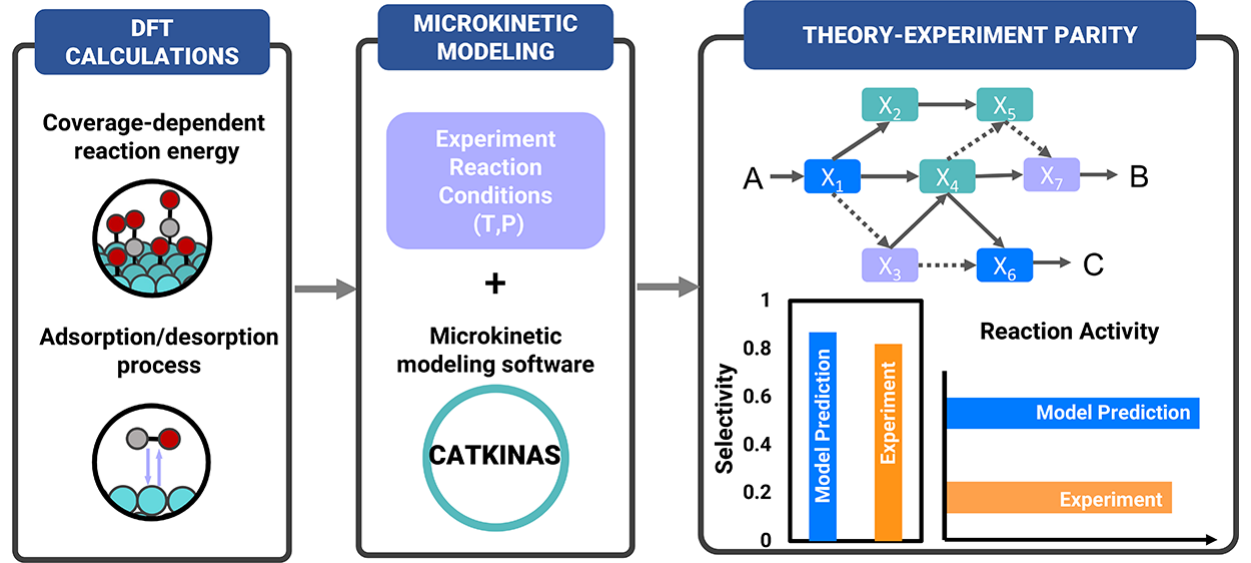

Microkinetic modeling based on density functional theory (DFT) energies plays an essential role in heterogeneous catalysis because it reveals the fundamental chemistry for catalytic reactions and bridges the microscopic understanding from theoretical calculations and experimental observations. Microkinetic modeling requires building a set of ordinary differential equations (ODEs) based on the calculation results of thermodynamic properties of adsorbates and kinetic parameters for the reaction elementary steps. Solving a microkinetic model can extract information on catalytic chemistry, including critical reaction intermediates, reaction pathways, the surface species distribution, activity, and selectivity, thus providing vital guidelines for altering catalysts.

However, the quantitative reliability of traditional microkinetic models is often insufficient to conclusively extrapolate the mechanistic details of complex reaction systems. This can be attributed to several factors, the most important of which is the limitation of obtaining an accurate estimation of the energy inputs via traditional calculation methods. These limitations include the difficulty of using static DFT methods to calculate reaction energies of adsorption/desorption processes, often rate-controlling or selectivity-determining steps, and the inadequate consideration of surface coverage effects. In addition, the robust microkinetic software is rare, which also complicates the resolution of complex catalytic systems.

In this Account, we review our recent works toward refining the predictions of microkinetic modeling in heterogeneous catalysis and achieving theory–experiment parity for activity and selectivity. First, we introduce CATKINAS, a microkinetic software developed in our group, and show how it disentangles the problem that traditional microkinetic software has and how it can now be applied to obtain kinetic results for more sophisticated reaction systems. Second, we describe a molecular dynamics method developed recently to obtain the free-energy changes for the adsorption/desorption process to fill in the missing energy inputs. Third, we show that a rigorous consideration of surface coverage effects is pivotal for building more realistic models and obtaining accurate kinetic results. Following a series of studies on acetylene hydrogenation reactions on Pd catalysts, we demonstrate how this new approach can provide an improved quantitative understanding of the mechanism, active site, and intrinsic structural sensitivity. Finally, we conclude with a brief outlook and the remaining challenges in this field.

## Key References

- ChenJ.; JiaM.; HuP.; WangH.CATKINAS: A Large-Scale Catalytic Microkinetic Analysis Software for Mechanism Auto-Analysis and Catalyst Screening. J. Comput. Chem.2021, 42, 379–39110.1002/jcc.2646433315262.^1^*An in-depth introduction of the microkinetic modeling software we developed and a demonstration of the efficient automicrokinetic analysis of multiscale catalytic systems with desired functions such as surface coverage determination and degree of rate control.*
- GuoC.; WangZ.; WangD.; WangH. F.; HuP.First-Principles Determination of CO Adsorption and Desorption on Pt(111) in the Free Energy Landscape. J. Phys. Chem. C2018, 122, 21478–2148310.1021/acs.jpcc.8b06782.^2^*In this work, an MD simulation-based free-energy approach was developed to investigate the adsorption/desorption process of CO on Pt(111) in order to resolve the controversy between experimental observations and the traditional calculation method. This is a robust approach to calculating the free-energy changes in the adsorption/desorption process.*
- GuoC.; MaoY.; YaoZ.; ChenJ.; HuP.Examination of the Key Issues in Microkinetics: CO Oxidation on Rh(1 1 1). J. Catal.2019, 379, 52–5910.1016/j.jcat.2019.09.012.^3^*In this work, we demonstrated that accurate kinetic results could be achieved via microkinetic modeling that includes a rigorous consideration of coverage effects on both adsorbates and transition states.*
- XieW.; XuJ.; DingY.; HuP.Quantitative Studies of the Key Aspects in Selective Acetylene Hydrogenation on Pd(111) by Microkinetic Modeling with Coverage Effects and Molecular Dynamics. ACS Catal.2021, 11( (111), ), 4094–410610.1021/acscatal.0c05345.^4^*In this work, a coverage-consistent microkinetic modeling approach that combined DFT calculation and AIMD with umbrella sampling was developed and utilized to elucidate full characterization of the reaction kinetics of acetylene hydrogenation on Pd(111) quantitatively.*

## 1 Why Do We Do Microkinetic Modeling?

Microkinetic modeling made its appearance by being able to reveal quantitatively the fundamental surface chemistry that controls catalyst performance.^5−7^ Quantum chemistry calculations have shown several unique advantages in studying heterogeneous catalytic reaction mechanisms at the atomic level, such as providing detailed information on the surface geometries, electronic structures, and energy barriers for elementary reactions steps. Among this atomic-scale information, direct comparisons of the reaction barriers are often used in explaining catalytic behavior, but the sheer quantity of this information is not enough on its own to solve the more significant problem of formulating a rational understanding of the reaction mechanism. This information alone cannot be compared with experimental observables, such as the reaction rates, turnover frequency (TOF), and selectivity.

Microkinetic modeling, using energies obtained by density functional theory (DFT) calculations, is often employed to bridge the gap, providing insights into the underlying mechanisms of heterogeneous catalytic reactions. Such microkinetic calculations are in principle entirely *ab initio*, which grants them the ability to mimic and predict macroscopic reaction kinetic results under experiment conditions while providing comprehensive and quantitative conclusions regarding reaction mechanisms. Early works by Norskov and co-workers have shown that insights from microkinetic modeling can serve as the basis for identifying new material compositions and atomic-scale architectures with improved catalytic activity and selectivity.^8−12^ To build a reliable microscopic kinetic model, an accurate estimation of the energy inputs is required. However, there are several major caveats of the traditional approaches to energy calculations, causing a lack of quantitative accuracy vis-à-vis experimentally obtained reaction kinetics: (i) To improve the model prediction, it is imperative to have a comprehensive and accurate set of energy inputs. However, the traditional calculation method has certain limitations. For example, it is difficult to accurately calculate the energetics of the adsorption/desorption process, which may be the rate-determining step or the key to elucidating product selectivity. (ii) Experimentally measurable reaction rates and TOF values are also closely related to surface coverages. Previous works by Li and co-workers and Norskov and co-workers have proven that a lack of consideration of surface environment effects, i.e, adsorbate-induced coverage effects, fails to describe the complex catalytic reactions.^11,13−15^ The resulting model predictions often show a mismatch of several orders of magnitude when compared to experimental data.^3,4,16,17^ Hence, a more comprehensive consideration of coverage is desired to align the model more closely to experimental conditions. Furthermore, there is still room for improvement in the current microkinetic modeling package when dealing with multiscale or complex systems.^1,18,19^

In this Account, we summarize our recent works on achieving theory–experiment parity in heterogeneous catalysis via mean-field microkinetic modeling. Specifically, we introduce a robust multiscale microkinetic modeling software, CATKINAS, developed by our group, which is a free-energy-based framework for obtaining free-energy barriers of the adsorption/desorption process, and a simply approach to incorporating a coverage effect to yield kinetic results with a more physically accurate description of catalytic systems. Subsequently, we show how to develop a generic strategy for building detail-rich microkinetic models. We present the general ideas and working principles of this strategy and demonstrate it with an inclusive example of selective acetylene hydrogenation on Pd(111). We demonstrate that good agreement can be achieved with the experiments regarding activity and selectivity. Furthermore, we explore the application of this approach to a quantitative understanding of the mechanism, active site, and intrinsic structural sensitivity of acetylene hydrogenation over Pd catalysts. Drawing on these examples, we strive to convey the advantages of a systematic and integrated approach that combines coverage-dependent DFT calculations, *ab initio* molecular dynamics (AIMD) simulations, and microkinetic modeling to incrementally improve the atomic-scale picture of important heterogeneous catalytic reactions.

## 2 CATKINAS: Multiscale Catalytic Microkinetic Analysis Software

One of our research foci has been developing next-generation microkinetic analysis software. In the early stage, microkinetic models were built in a case-by-case fashion, and numerical methods were utilized to solve specific problems.^20,21^ Recently, several microkinetic modeling programs were developed to break the limitations, including CatMAP, mkmcxx, and Micki.^22−24^ Each of these programs has its own attributes and has proven useful in many systems. However, concerns such as the stiffness problem of solving ordinary differential equations (ODEs) in more complex systems remain challenging.^18,19^

**Figure 1:**
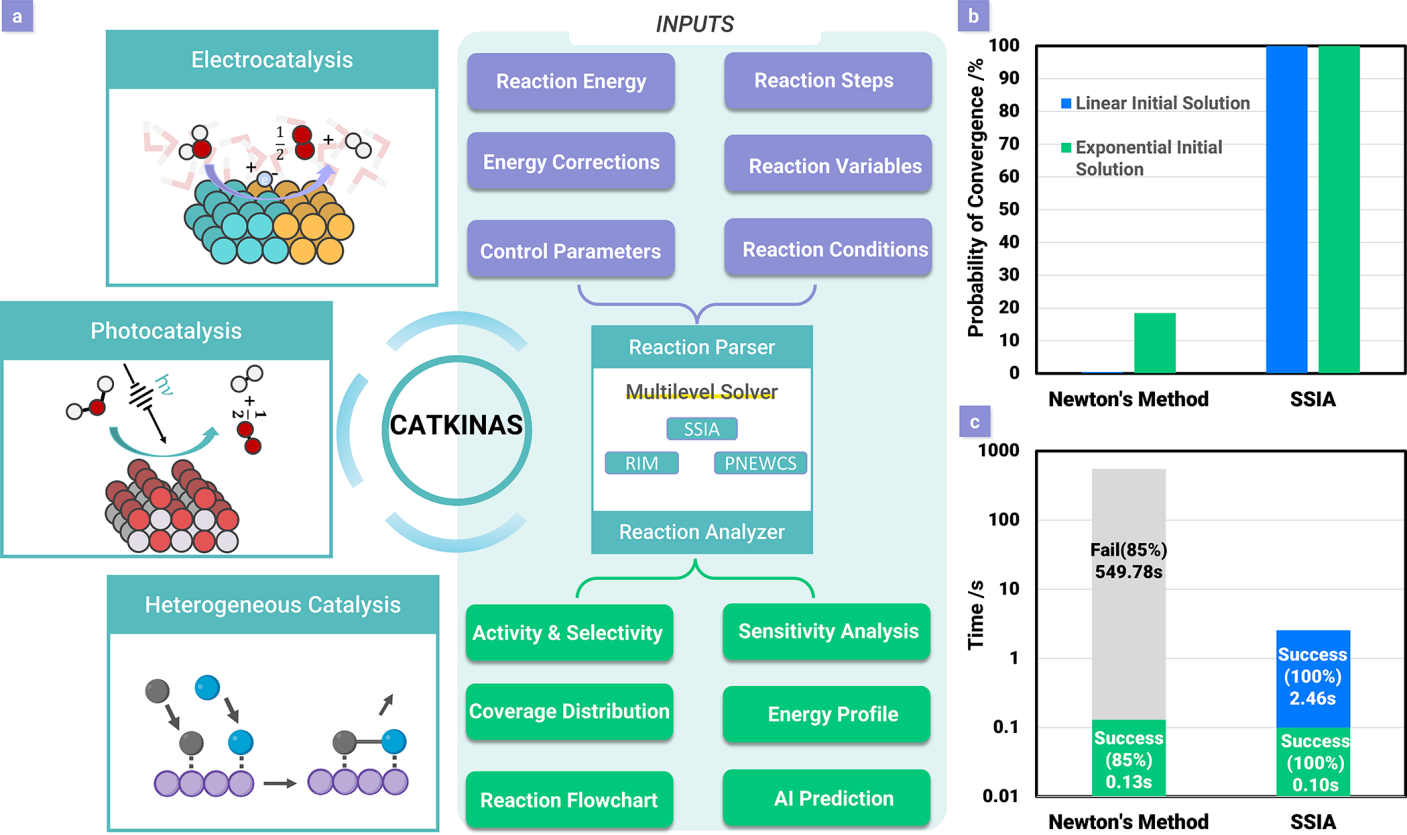
(a) Introduction of CATKINAS, including the multilevel solver that ensures quick and high-reliability convergence. (b) Convergence probabilities of the traditional damped Newton method and the SSIA method starting from linear initial solutions and exponential initial solutions.^18^ (c) Average time used and successful rate for solving the ammonia synthesis system by the traditional damped Newton method and the SSIA.^18^ Adapted with permission from ref (18). Copyright 2021 AIP Publishing.

CATKINAS (Catalytic Microkinetic Analysis Software, accessible at https://www.catkinas.com), as shown in Figure 1a, is multiscale catalytic microkinetic modeling software designed for automated reaction mechanism analysis and catalyst screening. Developed by our group, it has already demonstrated its power in building complex microkinetic simulations of catalytic reactions, and it satisfies the need for robustness and accuracy. Here we present a succinct introduction of CATKINAS’s advantages, including the following. (i) It possesses a multilevel solver in the core module of CATKINAS, which integrates multiple novel root-finding algorithms (SSIA,^18^ PNEWCS, and RIM^19^) and invokes these methods in a sequence to ensure the accuracy and speed of solving ODEs to achieve steady-state results. The SSIA algorithm developed by our group disentangles the problems associated with microkinetic software based on a modified Newton method, which rely excessively on initial guesses and often fail to converge for complex systems, or a pure ODE time-integration method, which is extremely memory-intensive and time-consuming because of the small step size.^18^ The efficiency of SSIA is benchmarked against Newton’s method in Figure 1b, where the convergence ability of both methods was tested with 1000 linear and 1000 exponential initial coverages for the CO oxidation reaction, and Figure 1c, where the solution time of both methods was tested with 100 sets of initial coverages from ammonia synthesis. (ii) The embodied result analysis function in CATKINAS, a variety of sensitivity analyses such as DRC,^25^ can be performed from multiple perspectives to identify which step, coverage of the adsorbants, and reaction conditions have the most impact on the total reaction rate, providing a direction for the optimization of the catalyst’s performance. (iii) It is user-friendly. CATKANS is out-of-the-box-style software with instant usability that only requires users to adjust the input file to describe their reaction of interest. In addition, automatic data visualization, including the reaction rate, surface coverage distribution, reversibility, reaction network, and energy profile, provides a better understanding of the microkinetic process and results.^1^

Our group has used CATKINAS to elucidate many vital catalytic reactions including photocatalysis,^26,27^ electrocatalysis,^28,29^ heterogeneous catalysis,^3,4,16,17^ and advancing catalysis theory and to provide more in-depth insights into experimental observations.

## 3 Key Issues in Improving the Accuracy of Microkinetic Model Predictions

**Figure 2:**
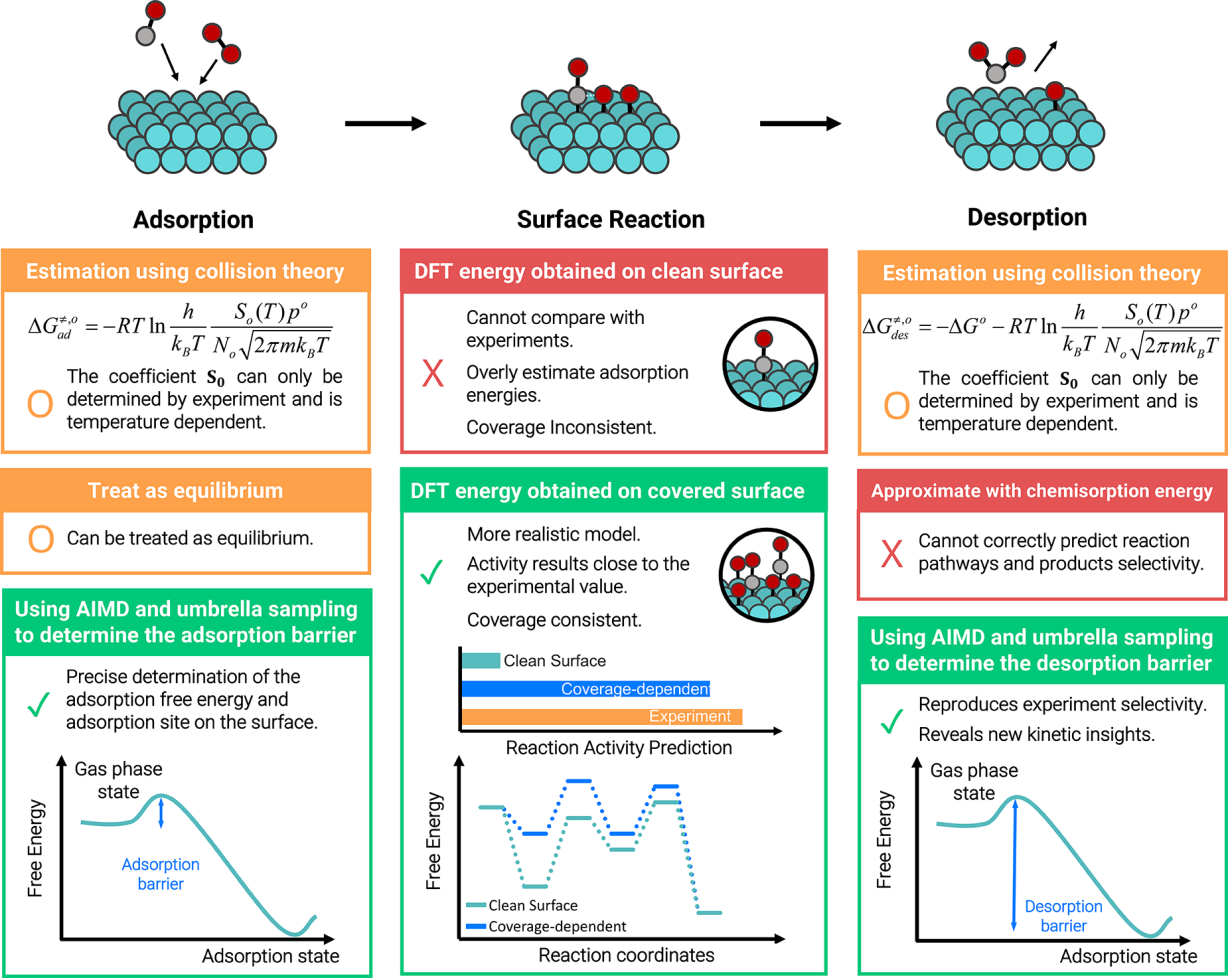
Schematic representation of the reaction steps in a general reaction in heterogeneous catalysis and the three key approaches our group adapted (green) in building a microkinetic model, including solving the issues of reaction barriers in the adsorption/desorption steps and coverage effect in surface reaction steps. Some microkinetic results from different approaches are listed.

Reactions in heterogeneous catalysis can be generalized to adsorption, surface reaction, and desorption, as shown in Figure 2. The key to formulating a conclusive microkinetic model is to obtain accurate first-principles energy inputs including adsorption/desorption energies and reaction barriers. Thus, improving the microkinetic model predictions requires more quantitative details from the elementary steps to achieve theory–experiment parity. In this Account, two recent works from our group, which are vital to adding more details to the microkinetic model and providing a better understanding of heterogeneous catalysis, are discussed below. The first one is the determination of adsorption/desorption barriers in a free-energy landscape, and the second one refers to how to better incorporate the coverage effect to restore a more realistic surface model and yield a quantitative description of reaction energies under realistic conditions.

### 3.1 First-Principles Determination of Adsorption and Desorption Processes

For traditional first-principles calculations, it is difficult to determine the reaction energy barrier of the adsorption/desorption process because entropies play an important role in the barrier. The traditional way of using the total energy with thermodynamic correction is based on approximations that ignore translational motions on the surfaces and can cause considerable errors.^2^ The Hertz–Knudesn equation based on collision theory was also adapted to approximate the adsorption/desorption barrier;^30^ however, the sticking coefficient from the equation needs to be determined experimentally for different surfaces and is highly temperature-dependent, which makes it difficult for general use. Because of the challenging nature of this problem, the adsorption process was habitually treated as an equilibrium^31,32^ and the desorption barrier was approximated as the chemisorption energy,^33^ which could lead to unpredictable errors, especially when the product selectivity was on the table because the desorption barrier often directly links to the selectivity analysis.

**Figure 3:**
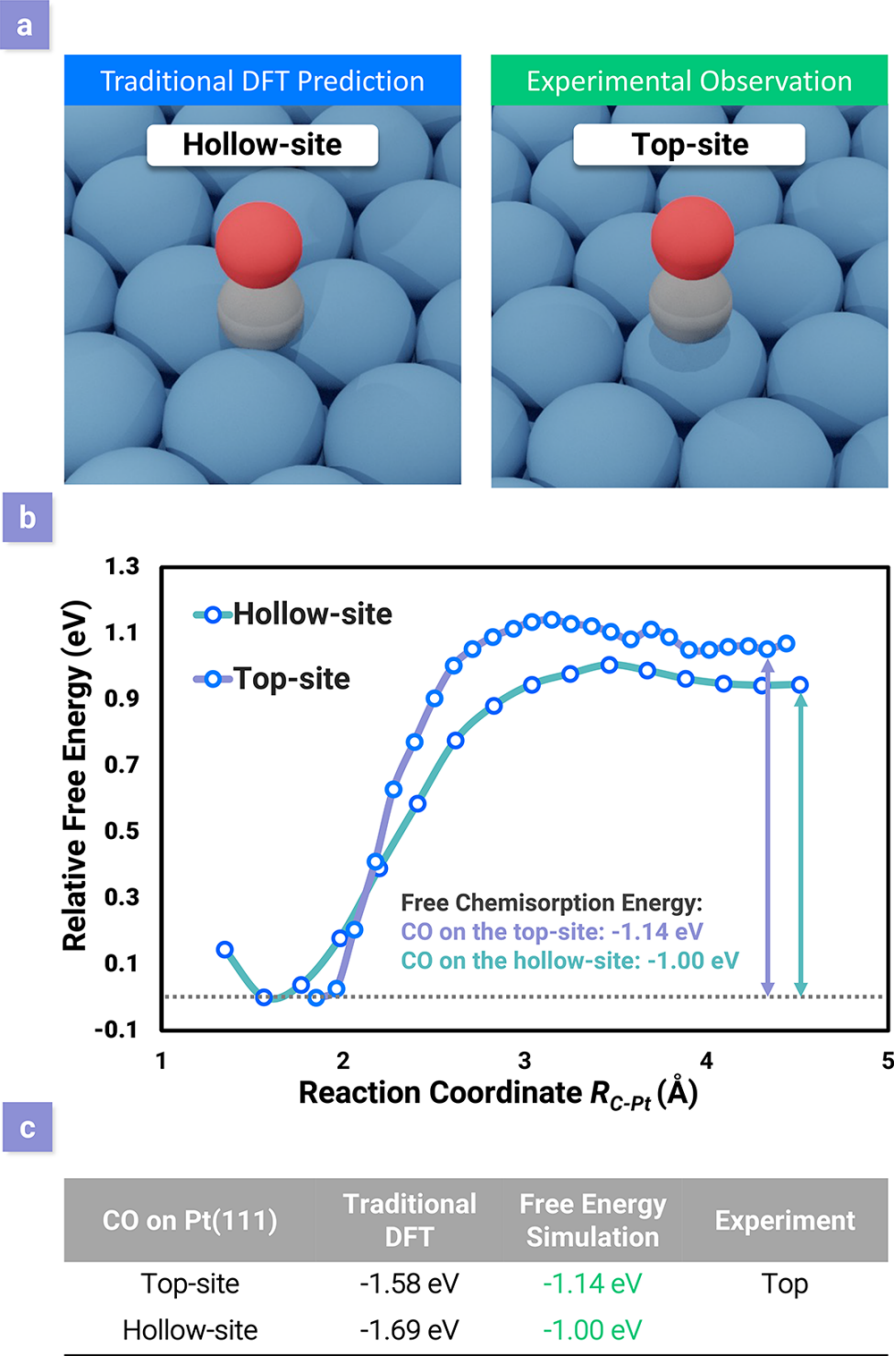
(a) “CO puzzle”, the controversy between the experimentally observed CO adsorption site (top site) of Pt(111) and the hollow site suggested by conventional DFT predictions. (b) Free energies of CO adsorption and desorption from the Pt top site (purple) and the hollow site (green) at a temperature of 300 K. (c) Comparison of the adsorption energies calculated from traditional DFT with thermodynamic adjustment and the free-energy simulation. The results from the newly developed AIMD method match the experimental observation. Adapted with permission from ref (2). Copyright 2018 American Chemical Society.

Our recent study illustrated the adsorption/desorption processes in the free-energy landscape using AIMD with umbrella sampling.^2^ Using the CO adsorption on Pt(111) as an example, we found that the results from the traditional total energy calculations might be substantially different from those from the free-energy-based simulations. Although CO adsorption on Pt(111) is one of the most studied catalytic systems and is the rate-determining step in a number of key reactions systems, there is still a long-standing debate on the adsorption structure. The traditional DFT calculation indicated that the adsorption energy on the hollow site is stronger than that on the top site, which is inconsistent with the experimental results. This is known as the “CO puzzle”.^34^ In previous works, the puzzle was solved when using a hybrid functional such as PBE0^35^ or adapting the random phase approximation (RPA)^34^ or the GGA+*U* approach.^36^ It is worth mentioning that these methods for obtaining the correct adsorption site are still static calculations performed at 0 K, but in a real system, all of the atoms are constantly vibrating, even at 0 K. Therefore, it is important to include surface atomic motion and enthalpy data in order to achieve more reliable results.^37^ The key characteristic of the AIMD with umbrella sampling method is that by including the atom motions it can directly give rise to the free energy at each reaction coordinate along the adsorption process, and it takes statistical fluctuations into consideration. One of the advantages of the MD simulation-based approach is that the temperature effect is naturally incorporated; we can map out the energy change in the free-energy landscape and thus obtain the adsorption/desorption energy instead of using a static calculation with thermodynamic adjustments. Furthermore, the results allow us to calculate the energy barrier of the adsorption/desorption from the detailed changes in free energy. In the CO adsorption example, the free-energy analysis reveals that CO prefers to adsorb on the top site (−1.14 eV free chemisorption energy) rather than on the hollow site (−1.00 eV), which is consistent with experimental observations as shown in Figure 3. Notably, this new method of obtaining the barriers can be easily applied to other systems, especially to those involving the liquid phase.^38−40^ In addition, we will elaborate more on how this method was used to help achieve theory–experiment parity for the C_2_H_4_ selectivity in the example of C_2_H_2_ hydrogenation on Pd in Section 4.

### 3.2 Surface Coverage Effect

**Figure 4:**
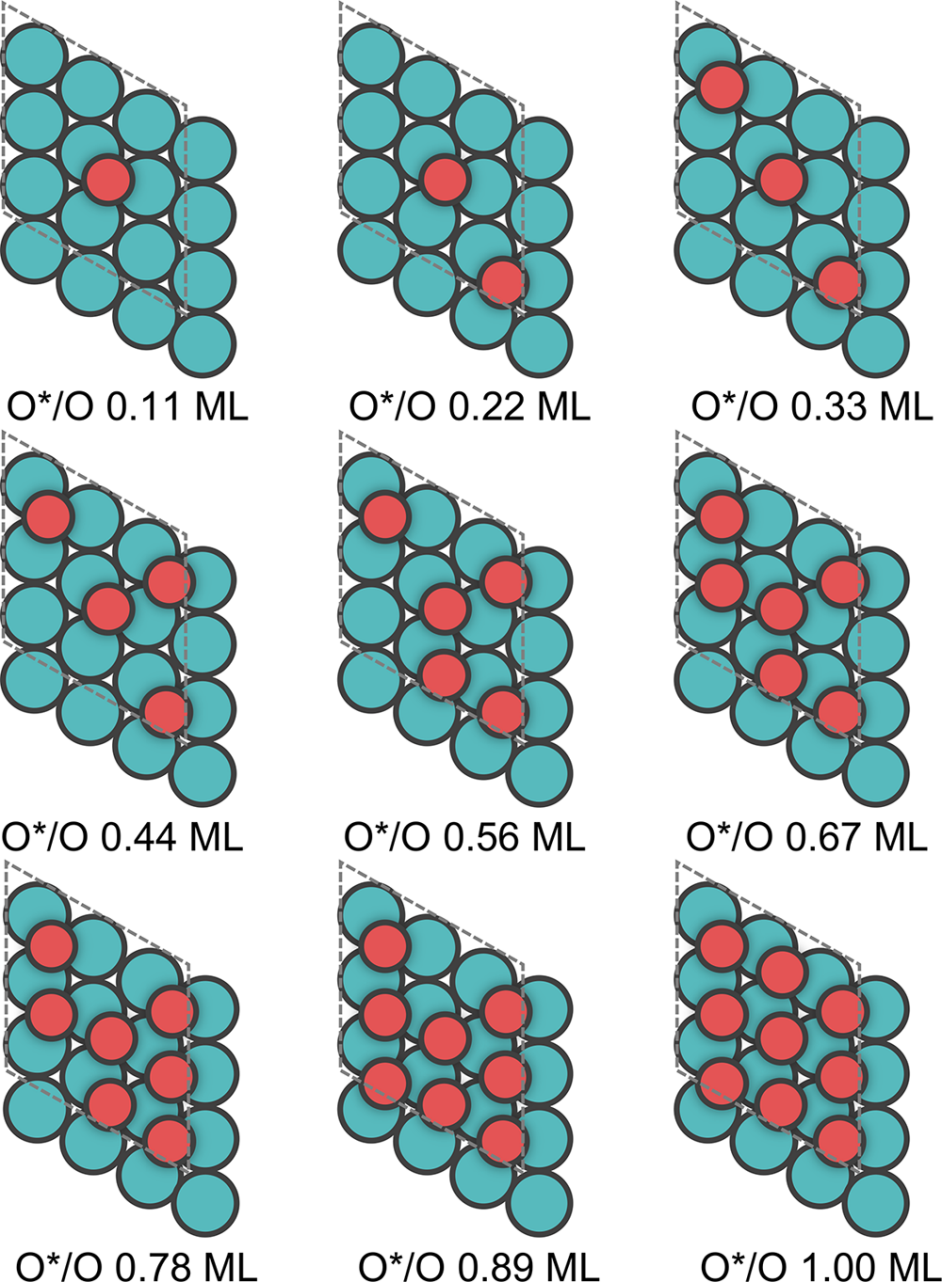
Structural illustrations of calculating the adsorbate–adsorbate self-interaction of O on a (3 × 3) Rh(111) surface at coverages from 0.11 to 1.00 ML.

The other challenge to improving the microkinetic prediction is to obtain accurate DFT energy inputs for surface reaction steps, as shown in Figure 2. Energy calculations conducted on a clean surface with only a partial or no surface environment considered may not fully capture the complexity of the real reaction.^41−43^ Although the resulting microkinetic model can have sound qualitative predictability, it is only a half-way solution to obtaining theory–experiment parity because of its intrinsic limitation. The fundamental issue of the surface coverage effect was examined for the example of the CO oxidation reaction on Rh(111).^3^ The TOF calculated in the previous theoretical study was around 10^–1^ s^–1^ using microkinetic modeling without considering the coverage effect, which is a few orders of magnitude lower than the experimental results of 10^2^–10^3^ s^–1^.^44^ In this work, the coverage effect was thoroughly studied, and the self- and cross-adsorbate–adsorbate interactions were calculated for both the adsorbates and transition states. In general, for each coverage and each type of interaction, all possible configurations were calculated, and the structure with the lowest energy will be used to establish the linear relationship between the coverage effect and chemisorption energies. An example of the self-adsorbate–adsorbate interaction of O is illustrated in Figure 4.

Furthermore, to achieve qualitative reconciliation, we have included several energy corrections: (i) energies derived from DFT calculations are at 0 K in temperature, and a thermodynamic correction need to be included to meet the experimental conditions;^45,46^ (ii) VASP DFT-PBE calculations do not perform well for gas-phase energies;^36,47^ therefore, all of the gas-phase molecules are calculated using Gaussian with B3LYP and the 6-311G basis set; and (iii) intermolecular interactions such as the van der Waals forces may need to be considered.^46,48^ We have made these adjustments and examined their effects on the theoretical reaction rates obtained by the microkinetic modeling compared with the experimental value, and we have concluded that the coverage effect is one of the most pivotal issues for obtaining accurate kinetic results. If both the self- and cross-interactions for adsorbates/transition states were considered, the TOF of CO oxidation on Rh(111) was calculated to be 3.2 × 10^3^ s^–1^, which is only 1 order of magnitude higher than the experimental results. With the additional corrections added to the microkinetic modeling, the TOF was calculated to be 8.2 × 10^2^ s^–1^, which is very close to the experimental result of 5.6 × 10^2^ s^–1^. This work laid an essential foundation for our subsequent studies of coverage effects on various complex reaction systems.

## 4 Case Study: Selective Acetylene Hydrogenation on Pd Catalysts

**Figure 5:**
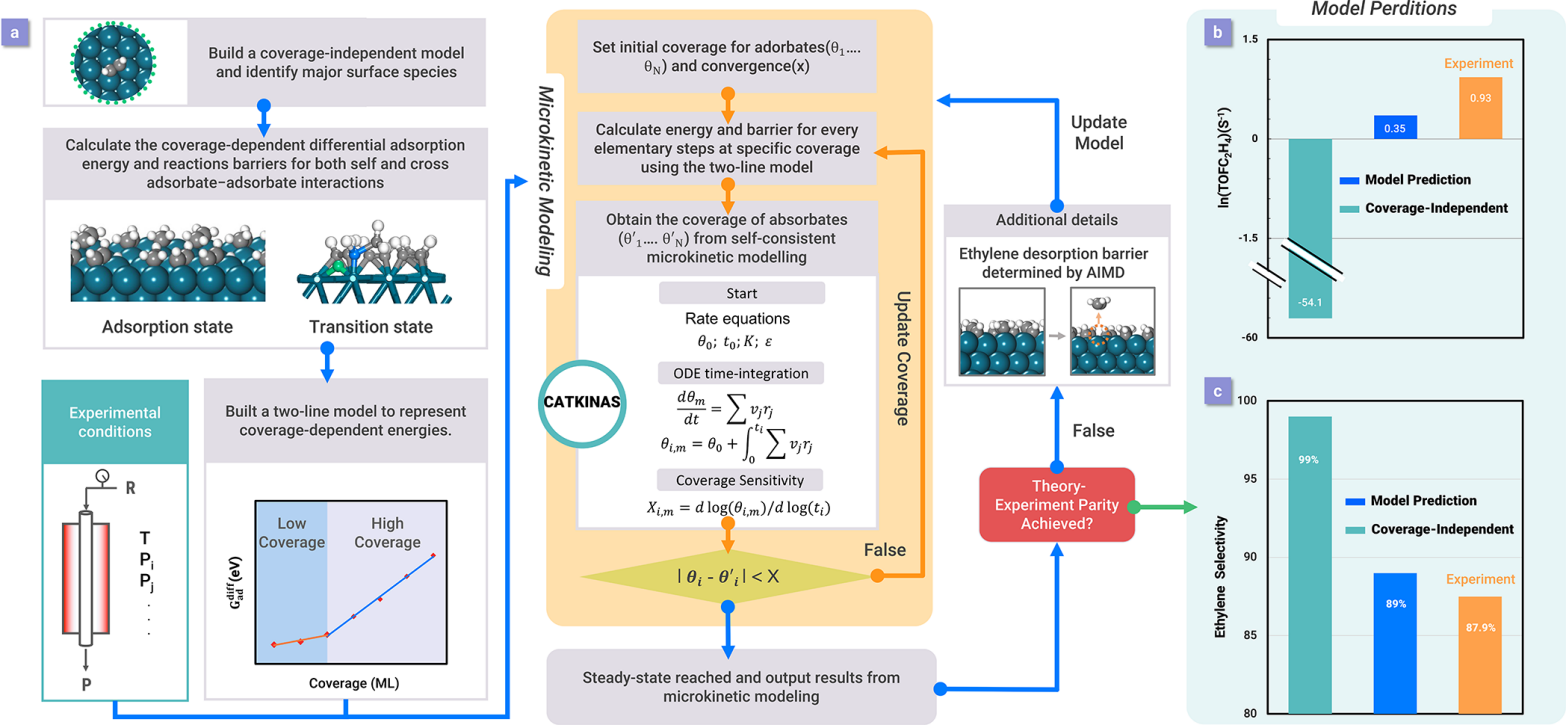
(a) Schematic representation of the workflow adopted in our self-consistent and coverage-dependent microkinetic modeling approach to achieving theory–experiment parity on selective acetylene hydrogenation on Pd(111). Information from the coverage-independent model was used to select the major surface adsorbates for calculating coverage-dependent energies. Each self- and cross-interaction between an adsorbate and major adsorbates was calculated. An iterative approach was used with CATKINAS to reach steady state. The desorption barrier of ethylene, a key reaction step that is hard to calculate with traditional methods, was determined by AIMD with umbrella sampling, which adds more critical details to our kinetic model. (b) TOF results of ethylene formation and (c) ethylene selectivity based on the coverage-independent model, coverage-dependent model, and comparison with the experimental value at 300 K. Adapted with permission from ref (3). Copyright 2021 American Chemical Society.

Hydrogenation reactions are among the most important classes of reactions, of which the hydrogenation of acetylene to ethylene is an important one with many applications, for example, in the production of polymers. The reaction occurs via a Horiuti–Polanyi mechanism, where C_2_H_2_ adsorbs first and is sequentially hydrogenated on metal surfaces. Herein, we present an overarching example of our approach to quantitively examine the catalytic performance of Pd catalysts on acetylene hydrogenation, starting with a coverage-independent model, progressing to a coverage-dependent model, and calculating the key desorption steps with AIMD to obtain a microkinetic model that achieves theory–experiment parity. A schematic representation of the workflow we used to investigate the acetylene hydrogenation on Pd(111) is shown in Figure 5.

### 4.1 Coverage-Dependent Microkinetic Model

For complex reactions, calculations of all of the cross-interactions between the transition states and different adsorbates, to consider the coverage effects as thoroughly as previously mentioned, would require extensive computational and time costs. However, the microkinetic results are predominantly affected by the most abundant surface adsorbates, and once these have been identified, we can conduct coverage-dependent calculations between all adsorbates and major adsorbates for an efficient representation of the surface coverage effect. One feasible way to identify the major adsorbates is to perform a coverage-independent analysis. As seen in Figure 5b, the coverage-independent microkinetic simulation results in a wholly poisoned surface and a TOF value that is many magnitudes lower than the experimental one. This means that the overly adsorbed C_2_H_2_ entirely blocks the reaction, suggesting that C_2_H_2_ will become one of the main adsorbates on the surface. From the coverage-independent energy profile in Figure 5a, C_2_H_3_ exhibits a larger adsorption energy, and the second hydrogenation step is kinetically hindered, making it a candidate for a major surface adsorbate. In addition, the H atom, because of its relatively small atomic size and strong adsorption on the surface, will be considered to be the last major adsorbate in our coverage-dependent study, which is also consistent with what was observed experimentally.^49,50^

To perform coverage-dependent microkinetic modeling, we first introduced a two-line model to quantify how the coverage effect between adsorbates and major adsorbates affects the chemisorption energies and reaction barriers. The reason for using the two-line model is the linear nature of the differential chemisorption free-energy-coverage relation^11,14^ and distinct impact level in the low- and high-coverage regions.^4,16^ The two lines describe the influence of adsorbate–major adsorbate interactions at different surface coverages. Once all of the interactions are established using the two-line model, we can obtain the value of the differential chemisorption energy of the target adsorbate under any coverage with any arbitrary distribution of major adsorbates using the following equation
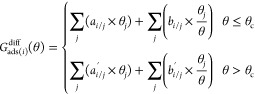
where θ, θ_*j*_, *i*, and *j* represent the total coverage, the coverage of adsorbate *j*, target adsorbate *i*, and major adsorbates *j*, respectively. *a* and *b* are the two parameters (slope and intercept) of the two-line model; *a* and *b* are used to describe the linear relationship in the low-coverage region, and *a*′ and *b*′ are for the high-coverage region. *a*_*i*/*j*_ is a measure of the extent to which the coverage of major adsorbates *j* affects the differential chemisorption energy of target adsorbate *i* if the coverage of *j* is increased. θ_c_ is the critical point separating the low coverage from the high coverage.

With the help of CATKINAS, a coverage self-consistent approach was adapted to achieve steady state for the reaction system. The iteration, shown in Figure 5, starts with a guessed initial distribution and updates the inputs depending on the convergence, determined by the difference between input and calculated coverage. Once all of the coverages have converged to a defined level, steady state is achieved. Using the self-consistent microkinetic model, the coverage-dependent TOF was calculated to be 1.4 s^–1^ [ln(TOF) = 0.35] at 300 K, which is very close to the experimental result of 2.66 s^–1^ [ln(TOF) = 0.98], shown in Figure 5b. The total surface coverage at steady state was 0.79 ML, containing 0.26 ML of C_2_H_2_, 0.34 ML of C_2_H_3_, and 0.19 ML of H, and the coverage of free sites was 0.21 ML, which is a more reasonable result. The microkinetic analysis also shows that the most abundant adsorbate on Pd(111) is C_2_H_3_ because the chemisorption free energy of C_2_H_3_ is comparatively higher than others under high-coverage conditions, and the C_2_H_3_ hydrogenation is unfavored kinetically.

This example illustrates the importance of incorporating the adsorbate–adsorbate interactions for adsorption and thoroughly calculating the adsorbate–transition state interactions and using the self-consistent coverage microkinetic model to bridge theoretical results with experimental data. In this step, the desorption process has been treated as equilibrium. To provide more rigorous kinetic results, particularly in terms of selectivity, an accurate calculation of the free-energy desorption barrier is critical.

### 4.2 First-Principles Determination of the C_2_H_4_ Desorption Process

As Studt et al. reported, to achieve the excellent selective hydrogenation of acetylene, the desorption barrier of ethylene for an ideal catalyst should be smaller than the hydrogenation barrier of ethylene.^51^ In the traditional model, the desorption process had been treated as occurring at equilibrium or has been estimated using the chemisorption energy as described in Section 3.1; however, both attempts lead to unreliable kinetic results compared to the 87.9% experimental ethylene selectivity on Pd(111) achieved by Li et al.^24^ The desorption barrier is not explicitly included if the process is estimated to occur at equilibrium, and incorporating this result into the microkinetic model developed in Section 4.1 leads to very high ethylene selectivity (99.7%). The second approach approximates the chemisorption energy of ethylene (−1.0 eV) as the reaction barrier; such a high desorption barrier tends to drastically shift the overall reaction in the direction of sequential hydrogenation, resulting in a less than 10% ethylene selectivity. Therefore, the ethylene desorption barrier is determined to be key to delivering more rigorous selectivity results and need to be accurately calculated.

In this work, the desorption barrier of ethylene was determined by AIMD with umbrella sampling as described in Section 3.1, which adds more critical details to the microkinetic modeling.^39,40,52^ A series of MD simulations were conducted on the Pd(111) surface with the round-up surface distribution (e.g., on a 4 × 4 surface, two C_2_H_2_ molecules are placed on the surface to achieve a 0.25 ML coverage) obtained from steady state in Section 4.1. The umbrella sampling with the weighted histogram analysis was used to determine the free desorption barrier,^2,40,52^ and it was calculated to be 0.59 eV at 300 K with a surface coverage of 0.79 ML. After factoring in the newly obtained desorption barrier of ethylene, the selectivity result was calculated to be 89% by our microkinetic model, which is in good agreement with the experimental result of 87.9% under the same conditions, as shown in Figure 5c.^24^

Therefore, we have walked through our strategy of combining static information from the rigorously conducted coverage-dependent first-principles calculations and dynamic results derived from AIMD to fully capture reaction details for quantitative microkinetic analysis and to achieve theory–experiment parity. Moreover, techniques such as the degree of rate control and sensitivity analysis can be applied to this model to reveal significant mechanistic insights. For example, on the basis of the sensitivity analysis, both the desorption barrier of ethylene and the further hydrogenation barrier of C_2_H_4_* have significant impacts on the selectivity of ethylene, but the change in the ethylene desorption barrier was found to have a more pronounced impact.^4^

### 4.3 Structural Sensitivity of Pd Catalysts

Another interesting issue in the system is the structural sensitivity, namely, the catalytic performance of the different active sites in terms of activity and selectivity.^53^ The structural sensitivity of catalysts is one of the most fundamental issues in heterogeneous catalysis, and the activity/selectivity of the acetylene hydrogenation reaction is known to be structure-sensitive. In some cases, Pd was reported to exhibit considerable selectivity toward ethylene, while other studies showed that Pd primarily promotes the production of ethane. This may be a result of surface defects. In the work of Molero et al.,^54^ the ethylene selectivity on Pd was about 30%; in the work of Li et al.,^55^ the ethylene selectivity was 87.9% on a Pd surface with 95% Pd(111) and 5% Pd(100). These divergent performances of Pd-based catalysts suggest that there is significant room to improve the understanding of the fundamental factors that control the activity and selectivity of acetylene hydrogenation.^26^ However, previous theoretical studies were still limited to simple comparisons using the adsorption energy or reaction barrier to determine the selectivity of different active sites and were unable to provide quantitative analysis.

**Figure 6:**
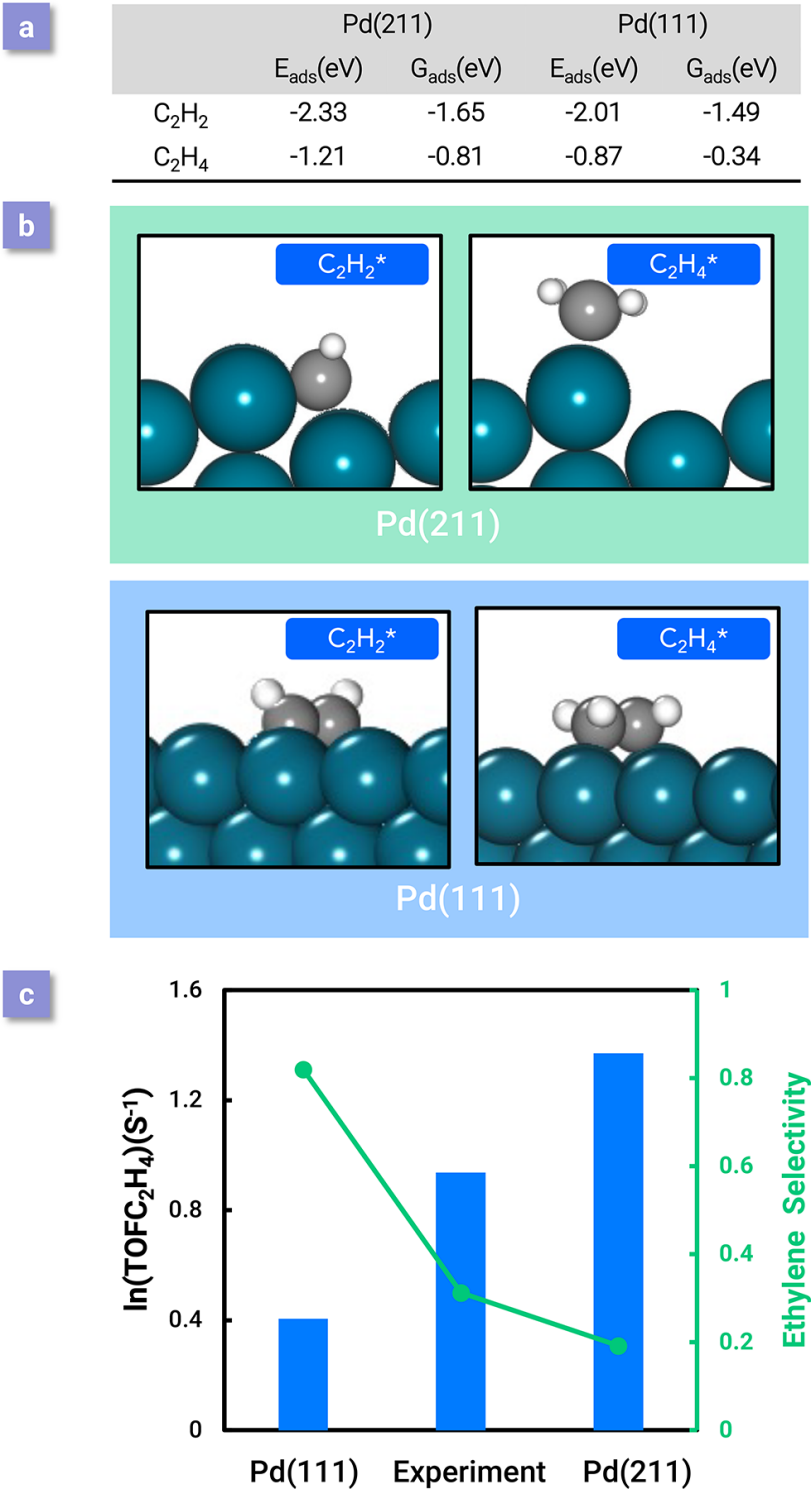
(a) Adsorption energies and free adsorption energies of C_2_H_2_ and C_2_H_4_ on Pd(211) and Pd(111). (b) Side views of C_2_H_2_ and C_2_H_4_ adsorption geometries on Pd(211) and Pd(111). (c) Comparison of the calculated TOF and selectivity results from the coverage-dependent microkinetic model and the experimental data from Molero et al.^54^ of ethylene production from acetylene hydrogenation over Pd(111) and Pd(211) at 300 K. Adapted with permission from ref (37). Copyright 2021 Royal Society of Chemistry.

With the confidence achieved by our coverage-dependent microkinetic modeling approach to the acetylene hydrogenation reactions on Pd(111) and motivated by the previous experimental differences, we further studied the structural sensitivity of Pd catalysts to provide a solid understanding of the structure effect on selectivity and activity at the atomic level. A rigorous coverage-dependent calculation was performed to obtain reaction energies, and AIMD was used to determine the desorption barrier of ethylene on Pd(211). It is found that both activity and selectivity toward ethylene are highly structurally dependent; our simulations show that Pd(211) is much more active than Pd(111), as shown in Figure 6c. On the other hand, the strong chemisorption energy limits the selectivity on Pd(211) (Figure 6a,c). After obtaining the self- and cross adsorbate–adsorbate interactions, TOF from the coverage-dependent model was calculated to be 3.9 s^–1^ [ln (TOF) = 1.37], which is higher than both the TOF from Pd(111) and the experimental results obtained from a Pd catalyst (which may contain different active sites). The desorption barrier of ethylene on Pd(211) was calculated to be 0.57 eV at steady state, although it was smaller than the desorption barrier (0.59 eV) of ethylene on Pd(111), and the selectivity toward ethylene (<20%) is much lower on the Pd(211) surface because of a significantly lowered C_2_H_4_* + H* ↔ C_2_H_5_*+* barrier. The microkinetic modeling suggests an almost inverse correlation between catalytic activity and selectivity based on the results from different active sites. The vastly different activity and selectivity results reported in the literature, even for catalysts that are nominally the same, can be rationalized as the variation in the active sites distribution because real catalysts will contain Pd(211)-like and Pd(111)-like surfaces. In addition, the overall coverage effects on Pd(211) are less influential than that on Pd(111) because of the geometric effect of the stepped surface; most adsorbates adsorb on the step edge on Pd(211), which makes them less impacted by the coverage effect, as can be seen in Figure 6b. However, because C_2_H_2_ adsorbs on the stepped B5 site, it is still heavily influenced by the coverage effect; an exclusive coverage dependence is necessary for obtaining quantitative results. Overall, this work on Pd(211) via microkinetic modeling proposed an atomic-level explanation for the differences in catalytic activity and selectivity reported in various studies in the literature and provided the opportunity to improve the performance of Pd-based catalysts, which are highly structure-dependent.

Using the case of selective acetylene hydrogenation on Pd catalysts, we demonstrated the strategy of developing a coverage-dependent microkinetic model formulated in our group. It speaks to the importance of developing coverage-dependent microkinetic modeling to decipher the essential surface chemistry involved in the catalytic reaction. Rigorously calculating the coverage effect and the adsorption/desorption process (i) yielded theory–experiment parity through theoretical and numerical methods and (ii) provided valuable insights into reaction pathways and determining key kinetic paraments and structural sensitivity of the reactions systems, which were critical to the understanding of the experimental results and reaction mechanism.

## 5 Perspectives and Conclusions

We have reviewed our comprehensive apporach that incorporates surface coverage effects in mcirokentic modedling to achieve theory-experiment parity. There are still many unexplored challenges and opportunities in the field of microkinetic modeling. The methodologies we developed have only been tested on model surfaces and that there is a “material gap” between simulations and experiment. The level of accuracy in the cases studies might not be general, partially due to error cancellations in the kinetic simulations when coverage effects are included. However, the mismatch in microkinetic modeling often vary by many orders of magnitude from experimental work if the aformentioned corrections like surface coverage effect and key adsorption/desorption process are not considered. We believe such approach have the potential to deliver improved quantitative accuracy in computational catalyst discoveray and reveal important mechanistic detials influencing catalystic performance. It is also worth noting that all of the microkinetic models mentioned in this Account are based on the mean-field approximation. Other formalisms such as kinetic Monte Carlo (KMC) simulations can also be adopted.^56−59^ The total rates obtained from the microkinetic models are based on the steady-state approximation, which may be different from the experimental kinetics that typically represent an integral over all stationary points of the reactor. Other selected challenges for further development in mcriokentic modeling are summarized here:

### 5.1 Complexed Surfaces

We are constantly trying to develop catalyst models that are better suited to the actual reaction conditions and obtain more accurate energy inputs for microkinetic modeling. Our works mentioned in this Account are primarily on the ideal monometallic surfaces, which are more conducive for us to focus on. We have added a few simple bimetallic alloys in our recent attempts at the direct synthesis of H_2_O_2_ over transition metals.^17^ Some of the methodologies mentioned here are yet to be employed in more sophisticated applications. More specifically, building plausible microkinetic models on metal oxides, designing single-site/atom catalysts, and constructing more complex metal–support interfaces (e.g., γ-Al_2_O_3_-supported catalysts)^100^ and nanoparticles with multiple active sites are yet to be investigated.

### 5.2 Improving the Accuracy of Energy Calculations

There are several ways to improve the quality of the involved first-principles calculations. One way of achieving better accuracy is by switching to more advanced functionals. Recent developments in functionals, e.g., vdW-DF2, BEEF-vdW, HSE06, RPA, etc., enable us to discern our position on Jacob’s ladder.^60−63^ Choosing the right one may substantially contribute to the energy calculations. For energy corrections, as mentioned in Section 4.2, the traditional quantum-harmonic approximations of correcting the total energy to the free energy failed to describe certain key reaction steps and may often underestimate the entropy of weakly bonded reactants, so methods such as AIMD can be adopted to obtain the free energy.^52^

### 5.3 Machine Learning

The utilization of machine learning seems to be a promising way to improve the inputs in microkinetic models.^64^ For example, calculations based on ideal surface models are often unable to represent the compositional variability and the associated complexity in these distorted amorphous structures. One possible way to solve surface reconstruction during reactions is to build potential energy surfaces (PES) via neural networks. Xu et al. proposed an on-the-fly machine learning method to accelerate AIMD simulations for adsorption energy estimations.^65^ Chen et al. applied the neural network potential and the genetic algorithm to identify the most stable configuration of Au@Pt, which is a common electrocatalytic system.^66^ Li et al. studied the Pd–Ag–H system for the acetylene hydrogenation reactions using the global neural network potential.^67^ Constructing an accurate PES can be time-consuming and resource-intensive, but the rapid development of machine learning has provided a possible pathway for building microkinetic models on more complex or amorphous surfaces.

In this Account, we demonstrated the approach of using CATKINAS with coverage-dependent energy calculations and an advanced simulation method such as AIMD; we can unleash the full potential of microkinetic analysis even on complex reaction systems. Moreover, by providing a qualitative determination along with accurate quantitative metrics, this methodology can percolate into the process of catalyst screening and can be applied to other systems in heterogeneous catalysis to guide the rational design of novel catalysts.
